# A Hospital for Europeans at Hongkong

**Published:** 1905-04-29

**Authors:** 


### A HOSPITAL FOR EUROPEANS AT HONGKONG.

To the already rather numerous hospitals in Hongkong another will shortly be added. Its fabric is rapidly approaching completion, and the building should be ready for occupation before the end of the present year. The institution, which, according to the wish of the donor, is to be named "The Matilda Hospital," in memory of his wife, is situated on the shoulder of Mount Kellett, in the Peak District, and will be an extensive and well-fitted, if not an actually imposing structure. It is to cost about £35,000, and is being very strongly built of the best materials, steel, and concrete, teak and cut granite being freely used. It will provide accommodation for a large number of patients, and there will be quarters for the doctors and nurses in separate blocks, though in the same compound. This hospital is being erected by the executors of the late Mr. Granville Sharp, formerly a banker, and more recently an estate agent, who spent the greater portion of a long life in the colony and amassed a considerable fortune. Mr. Granville Sharp's wife predeceased him, and he left no children. By his will, after bequeathing legacies to his brother and other relatives, he directed that the remainder of his estate should be devoted to the erection and endowment of a hospital for the benefit of those needing such acccommodation who were not of Chinese or Portuguese blood. He also stipulates that drugs shall be very sparingly used, and he originally wished to insist that the medical services should be gratuitously given until it had been pointed out to him that this would be both unreasonable and unworkable, as the local doctors maintain a hospital at the Peak at their own risk and for their own patients, and could not be expected to give their services to an establishment which might in a manner come into competition with them. The property left by Mr. Sharp has doubled in value since his decease, and the relatives, thinking that as the executors had provided for the erection of a hospital on the lines laid down by the testator, and also for its adequate endowment, it might be within the power of the Supreme Court to make an order for the application of the balance of money accruing from appreciation of the property for their use and benefit, instituted a suit for the purpose. The Chief Justice, however, was unable to make any such order, for, as he pointed out, it was clearly laid down in the will that, if any donations or bequests were offered to the hospital they were on no account to be refused, even if the sum left were found fully adequate for the expenses. Clearly it was in the mind of the testator that the last penny of his estate should be devoted towards the great object he had in view. A costly and commodious building which, owing to the eccentric conditions of the will, must necessarily be more of a convalescent home than a hospital is therefore to be at the disposal of the more indigent of the European population of the Colony and the China Treaty ports.

